# Identifying Transportation Needs in Ophthalmology Clinic Notes Using Natural Language Processing: Retrospective, Cross-Sectional Study

**DOI:** 10.2196/69216

**Published:** 2025-09-05

**Authors:** Lauren M Wasser, Hai-Wei Liang, Chenyu Li, Julie Cassidy, Pooja Tallapaneni, Hunter Osterhoudt, Yanshan Wang, Andrew M Williams

**Affiliations:** 1 Department of Ophthalmology, University of Pittsburgh School of Medicine, 1622 Locust Street, 5th floor, Pittsburgh, PA, 15219, United States, 1 412-642-5382; 2 Department of Health Information Management, University of Pittsburgh School of Health and Rehabilitation Sciences, Pittsburgh, PA, United States

**Keywords:** social needs screening, electronic health record, social determinants of health, transportation needs, natural language processing

## Abstract

**Background:**

Transportation insecurity is a known barrier to accessing eye care and is associated with poorer visual outcomes for patients. However, its mention is seldom captured in structured data fields in electronic health records, limiting efforts to identify and support affected patients. Free-text clinical documentation may more efficiently capture information on transportation-related challenges than structured data.

**Objective:**

In this study, we aimed to identify mention of transportation insecurity in free-text ophthalmology clinic notes using natural language processing (NLP).

**Methods:**

In this retrospective, cross-sectional study, we examined ophthalmology clinic notes of adult patients with an encounter at a tertiary academic eye center from 2016 to 2023. Demographic information and free text from clinical notes were extracted from electronic health records and deidentified for analysis. Free text was used to develop a rule-based NLP algorithm to identify transportation insecurity. The NLP algorithm was trained and validated using a gold-standard expert review, and precision, recall, and *F*_1_-scores were used to evaluate the algorithm’s performance. Logistic regression evaluated associations between demographics and transportation insecurity.

**Results:**

A total of 1,801,572 clinical notes of 118,518 unique patients were examined, and the NLP algorithm identified 726 (0.6%) patients with transportation insecurity. The algorithm’s precision, recall, and *F*_1_-score were 0.860, 0.960, and 0.778, respectively, indicating high agreement with the gold-standard expert review. Patients with identified transportation insecurity were more likely to be older (OR 3.01, 95% CI 2.38‐3.78 for those aged ≥80 vs 18‐60 y) and less likely to identify as Asian (OR 0.04, 95% CI 0‐0.18 for Asian patients vs White patients). There was no difference by sex (OR 1.13, 95% CI 0.97‐1.31) or between the Black and White races (OR 0.98, 95% CI 0.79‐1.22).

**Conclusions:**

NLP has the potential to identify patients experiencing transportation insecurity from ophthalmology clinic notes, which may help to facilitate referrals to transportation resources.

## Introduction

Health-related social needs (HRSN) affect access to eye care [1], and transportation needs in particular are a commonly cited barrier to accessing care for chronic eye diseases, such as diabetic retinopathy and glaucoma [2,3]. For instance, among respondents to the National Health Interview Survey, lack of transportation was associated with delayed follow-up with an ophthalmologist or optometrist, and transportation-related barriers to care disproportionately affected respondents with glaucoma or vision loss [4]. Separately, telephone interviews with patients with glaucoma following appointment no-show revealed that transportation was commonly cited as the reason for a missed appointment [5].

Fortunately, transportation needs are addressable, and referral to social work or patient navigator services can connect patients in need with medical transportation through local resources and programs [6-8]. At our own institution, the need for transportation to eye appointments is the most common indication for referral to our patient navigator program, and these transportation referrals are addressed successfully through engagement with local resources or by enrollment in patients’ insurance-sponsored medical transportation programs [8].

A barrier to connecting vulnerable patients to transportation resources is identifying which patients have a transportation need. Clinic-administered questionnaires to assess for HRSN are important screening tools, but survey uptake in clinical settings is suboptimal [9,10]. Furthermore, social determinants of health (SDoH), which encompass HRSN, are rarely available in structured electronic health record (EHR) data, such as diagnosis codes, and are more commonly documented in unstructured, narrative clinical notes [11-13]. Fortunately, unstructured notes may be accessible for analysis using technology such as natural language processing (NLP), an artificial intelligence (AI) tool that extracts and classifies free-text language [14]. In preliminary studies, NLP has demonstrated capability to identify SDoH terms from EHR text with reasonable accuracy compared to expert review [15,16].

The aim of this study was to determine whether NLP can be used to identify patients’ transportation needs from existing unstructured clinical notes in the EHRs of our academic ophthalmology practice.

## Methods

### Ethical Considerations

This retrospective, cross-sectional, observational study was approved by the University of Pittsburgh institutional review board (STUDY21040204), adhered to the tenets of the Declaration of Helsinki, and complied with the Health Insurance Portability and Accountability Act of 1996. Patient informed consent was waived by the institutional review board. Privacy and confidentiality protection was ensured by leveraging an honest broker to deidentify patient data before analysis, and all analyses were performed on secure university servers. No patient compensation was provided.

### Data Source

EHR data were obtained from outpatient clinical encounters of adult patients with the University of Pittsburgh Medical Center Department of Ophthalmology from January 2016 through February 2023. These data included unstructured free text from clinical notes, demographic information (including age, race and ethnicity, and sex), and semistructured text. Semistructured text included questionnaires in the social history section of physician and clinic staff notes, which indicated whether transportation needs were available on file. All signed clinical notes from an ophthalmology encounter were included, including office visit notes from technicians, trainees, and attending physicians, as well as documentation from telephone and refill encounters. These data were extracted from our institution’s EHR (Epic Systems) using a custom-made research data warehouse [17]. After extraction, the structured data were deidentified by an honest broker, who is an official certified by the health system and the university to remove identifying information from health records, including names, addresses, and phone numbers, before transferring the dataset to investigators. Free-text notes were deidentified by National Library of Medicine Scrubber [18]. For patients with multiple encounters over the study period, demographic information was included from the first visit.

### Algorithm Development

Due to the sparse documentation of transportation in encounter notes, we used a list of keywords (eg, “transportation” and “ride”) to sample a subset of notes that contain transportation-related information. We then randomly selected 300 notes to create a gold-standard dataset manually reviewed by 2 ophthalmologists (LMW and AMW) and a research assistant (JC). We used 70% as training data to develop the rule-based NLP algorithm, termed NLP4Eye, with the remaining 30% serving as validation data for evaluation. After removing duplicated notes, there were 188 notes in the training dataset and 106 notes in the testing dataset.

NLP4Eye was iteratively refined on the training dataset. Embedded text of standardized questionnaire items that included transportation language was filtered out, as were transportation-related terms that predominately referred to visual function rather than social need (eg, terms related to “driving” were frequently mentioned during cataract evaluation encounters and thus removed from the algorithm dictionary as indicators of a transportation need). Similarly, due to its frequent mention in the context of fluorescein angiography, “transit” was removed from the dictionary of transportation-related terms in the NLP algorithm.

The NLP algorithm uses these rules within detected sentences to identify transportation needs from notes. The rules are “\b(%reTransportation).(%reNeeds)\b” or “\b(%reNeeds).(%reTransportation)\b,” where “reTransportation” represents regular expressions for transportation-related words and “reNeeds” represents needs-related information, as detailed in Textbox 1. The NLP algorithm was then evaluated on the held-out testing data.

After the NLP algorithm development was completed, its validation was assessed on the testing dataset. The *F*_1_-score of the NLP algorithm was then calculated to determine its performance in relation to the gold-standard testing dataset. The *F*_1_-score, reported between 0 and 1, is a common metric of NLP performance relative to a reference standard. *F*_1_ is calculated as a harmonic mean of the algorithm’s precision (analogous to positive predictive value) and recall (analogous to sensitivity). An *F*_1_ value of 0.7 or greater was considered adequate.

Finally, we assessed the algorithm for potential false negatives by conducting an additional post-hoc evaluation. In this evaluation, we assessed 200 clinical notes prefiltered for the tokens “transport*” and “mobility restriction*.” These broader tokens were selected to capture any transportation-related mentions that might have been missed by our NLP algorithm, allowing us to evaluate potential false negatives in our algorithm’s performance. An ophthalmologist (AMW) then annotated these notes for transportation needs, and discrepancies between the algorithm and physician annotations were analyzed using the taxonomy for systematic error analysis in clinical concept extraction by Fu et al [19].

The NLP4Eye algorithm was developed using the Open Health Natural Language Processing Toolkit, an NLP tool based on the Apache Unstructured Information Management Architecture pipeline, to facilitate information extraction from clinical narratives [20]. We created rules for identifying transportation needs based on physicians’ knowledge and experience, refining these rules through physician verification. The regular expressions used in our NLP algorithm are listed in Textbox 1.

Textbox 1. **Expressions used by the NLP4Eye algorithm to identify transportation needs.**

- Transportation terms: “transportation,” “ride,” “driv(e|ing)”
- Transportation need terms: “work on,” “option(s),” “issue(s),” “due to,” “assist(ance)?,” “access,” “adequate,” “discuss(ed|es|ing),” “(un)?able,” “accommodation,” “support,” “agree(s)?,” “appl(y|ies),” “arrang(es|ed|e|ing)?,” “need(s),” “blam(e|es|ing),” “find(s|ing),” “connect(ing|ed)?,” “could(n’t| not),” “did(n’t|not),” “difficult(y|ies)?,” “get(ting)?,” “help,” “does(n’t| not),” “lack(s|ing)?,” “easier,” “EMS,” “investigate,” “us(es|e|ing),” “depend(s|ing)?,” “organiz(es|e|ing),” “coordination,” “possible,” “resources,” “limit(s|ations)?,” “convenience,” “trouble(s)?,” “secur(es|e|ing),” “concern(s)?,” “regarding,” “reli(es|able|ant),” “time allowance,” “problem(s)?,” “challeng(es|e|ing),” “purpose(s)?,” “offer(s|ed),” “provid(es|ed|e|ing)”

### Statistical Analysis

Statistical analyses were performed using R software (version 4.4.1; R Core Team). Descriptive statistics were used to summarize continuous variables as mean (SD), and categorical variables were presented as percentages and compared using chi-square tests (Fisher exact tests when cell values were less than 5). Longitudinal descriptive statistics were summarized as median (IQR) due to a violation of the normality assumption. Binary logistic regression analysis was conducted to identify predictors of NLP-identified transportation needs. Odds ratios (ORs) were calculated with 95% CIs, and a *P* value <.05 was considered statistically significant.

## Results

### Study Population

A total of 1,801,572 clinical notes from 118,518 unique adult patients were extracted from ophthalmology encounters over the study period, averaging 15.2 (SD 25.19) encounters per patient. Half (59,299/118,518, 50.0%) of the patient population was aged 60 years or above, 57.8% identified as female (68,485/118,518), and the racial composition was mostly White (90,699/118,518, 76.5%), followed by Black (17,594/118,518, 14.8%; Table 1).

**Table 1.** Demographic characteristics and their unadjusted associations with the identification of a transportation need by a natural language processing algorithm.

Characteristics	Total, n (%)	Transportation need identified, n (%)	No transportation need identified, n (%)	Odds ratio (95% CI)	*P* value
Age (years)					<.001
18 to <60	59,219 (50.0)	204 (28.1)	59,015 (50.1)	Reference	
60 to <70	28,434 (24.0)	179 (24.7)	28,255 (24.0)	1.83 (1.50-2.24)	
70 to <80	19,903 (16.8)	218 (30.0)	19,685 (16.7)	3.20 (2.65-3.88)	
>80	10,962 (9.2)	125 (17.2)	10,837 (9.2)	3.34 (2.66-4.17)	
Sex					.11
Female	68,485 (57.8)	441 (60.7)	68,044 (57.8)	1.13 (0.97-1.31)	
Male	50,032 (42.2)	285 (39.3)	49,747 (42.2)	Reference	
Race					<.001
Asian	5041 (4.3)	1 (0.1)	5040 (4.3)	0.03 (0-0.13)	
Black	17,594 (14.8)	104 (14.3)	17,490 (14.8)	0.88 (0.71-1.07)	
Native American and Alaska Native	300 (0.3)	2 (0.3)	298 (0.3)	0.99 (0.16-3.08)	
Pacific Islander	147 (0.1)	1 (0.1)	146 (0.1)	1.01 (0.06-4.50)	
White	90,699 (76.5)	612 (84.3)	90,087 (76.5)	Reference	
Declined or missing	4737 (4.0)	6 (0.8)	4731 (4.0)	N/A^a^	
Ethnicity					.01
Non-Hispanic or Latino	108,402 (91.5)	4731 (4.0)	107,707 (91.4)	4.99 (1.61-30.05)	
Hispanic or Latino	1548 (1.3)	2 (0.3)	1546 (1.3)	Reference	
Declined or missing	8568 (7.2)	29 (4.0)	8539 (7.2)	N/A	

a N/A: not applicable.

### NLP Algorithm Validation

In the training stage of NLP algorithm development, 176 out of 188 (93.6%) notes were determined to be correctly labeled by the algorithm. The most common contexts for the 12 false hits were confirmed transportation support (6/188, 3.2%; eg, “agrees to be his transportation to and from and assist with care”), mention of transportation as the setting for an ocular injury (2/188, 1.1%), typographical error (2/188, 1.1%; both instances included “transportation” in place of “transplantation” in the context of corneal grafting), and transportation in the wrong context (2/188, 1.1%; eg, cellular transportation as a target for drug delivery). The algorithm was further refined based on these reference data and implemented across the dataset.

In the validation stage of NLP algorithm development, 95 of 100 (95%) notes were confirmed to represent a transportation need. Of the 5 false positives, 2 were typographical errors in the notes (both meant to refer to eye muscle “transposition”), 2 referred to the Pennsylvania Department of Transportation (one for a required vision screening examination and one to state that the patient retired from that organization), and 1 confirmed reliable transportation. The NLP algorithm’s precision, recall, and *F*_1_-score were 0.860, 0.960, and 0.778, respectively, indicating high agreement with the gold standard expert review.

In addition to measuring our algorithm performance with the *F*_1_-score, we also assessed for false negatives or transportation needs that could be detected using broad transportation terms but later missed during the iterative development of the algorithm. Our post-hoc evaluation on 200 notes containing the tokens “transport*” and “mobility restric*” revealed additional insights about algorithm performance. Within this broader sample, our algorithm correctly identified 101 notes without true transportation needs and 70 of 99 (70%) notes with true transportation issues (accuracy 85.5%, precision 1.00, recall 0.71, and *F*_1_-score 0.83). Analysis of the 29 false negatives using the taxonomy by Fu et al [19] showed that approximately half represented contextual errors (primarily negation and possible or probable language) and half were linguistic errors (mainly synonym mismatches and implied inferences; additional details are available in Multimedia Appendix 1). These findings suggest that our reported prevalence of transportation needs likely represents a conservative estimate.

### NLP Identification of Transportation Needs

We applied the NLP algorithm to the entire cohort of 118,518 unique patients. The NLP algorithm flagged 726 (0.6%) unique patients as having at least one mention of a transportation need in their ophthalmic clinical notes. These 726 patients had a total of 1033 mentions of transportation needs, averaging 1.42 mentions per patient over the study period. As delineated in Table 2, the most common transportation-related terms identified by the NLP algorithm included the following phrases: “due to transportation,” “transportation issues,” and “transportation difficulties.”

Textbox 2 illustrates representative examples of sentences from clinical notes that provide context for the NLP search hits.

**Table 2.** Top 10 transportation-related terms identified by the NLP4Eye algorithm.

Term	Encounter hits, n	Unique patient hits, n
Due to transportation	171	121
Transportation issues	154	109
Difficulty with transportation	48	24
Transportation difficulties	19	9
Arrange transportation	16	14
Issues with transportation	10	6
Access transportation	10	9
Transportation options	9	6
Unable to get transportation	8	6
Needs transportation	5	1

Textbox 2. **Representative examples of sentences from clinical notes that were identified as expressing transportation needs by the NLP4Eye algorithm.**

**Example 1**

- Identified sentence: “She is unable to stay for an injection due to ride issues today and wishes to observe.”
- Regular expression: “due to ride issues”

**Example 2**

- Identified sentence: “He states that he never returned for any post-operative exams after the surgery in December due to transportation issues.”
- Regular expression: “due to transportation issues”

**Example 3**

- Identified sentence: “It was decided by the Pt to use Ozurdex today for [PERSONALNAME] but no ozrudex in the office today and she has no transportation.”
- Regular expression: “no transportation”

**Example 4**

- Identified sentence: “Huge change os, noticed 3 months ago, had appt in Aug. 2016, had to cx that appt., no ride.”
- Regular expression: “no ride”

### Demographic Characteristics Associated With NLP-Identified Transportation Needs

Compared to patients without an identified transportation need, patients who had at least one mention of a transportation need identified by the NLP algorithm were more likely to be aged above 80 years rather than below 60 years (adjusted OR 3.01, 95% CI 2.38‐3.78), and they were less likely to identify as Asian (adjusted OR 0.04, 95% CI 0‐0.18 for Asian patients vs White patients; Table 3). There was no difference between the female and male sexes (OR 1.13, 95% CI 0.97‐1.31) or between the Black and White races (adjusted OR 0.98, 95% CI 0.79‐1.22; Tables1  3).

**Table 3.** Adjusted associations between demographic characteristics and the identification of a transportation need by the NLP4Eye algorithm.

Characteristics	Odds ratio (95% CI)	*P* value
Age (years)		<.001
18 to <60	Reference	
60 to <70	1.71 (1.39-2.11)	
70 to <80	3.01 (2.47-3.67)	
>80	3.01 (2.38-3.78)	
Race		<.001
Asian	0.04 (0.00-0.18)	
Black	0.98 (0.79-1.22)	
White	Reference	
Ethnicity		.05
Non-Hispanic or Latino	3.11 (1.00-18.78)	
Hispanic or Latino	Reference	

### Longitudinal Analysis of Transportation Needs Over Time

Among patients with at least one documented transportation need, the average annual rate of transportation need mention per year from 2016 through 2022 ranged from 2.6% to 4.9%, with the lowest in 2017 and the highest in 2022 (Table 4). Regarding the number of documented transportation needs, most patients were identified only once (566/726, 78.0%), occurring for a median of 127 (IQR 21.3-595.3) days after their first encounter. Of the total, 20.0% (145/726) of patients had between 1 and 5 additional transportation needs, with a median time of 4, 69, 42, and 96 days between each respective event; 2.1% (15/726) of patients had more than 5 documented transportation needs, with the highest being 9 instances. Overall, the median time from the first visit to transportation-related events gradually increased over time, whereas the time between consecutive needs did not follow a consistent pattern (Table 5).

**Table 4.** Rates of transportation needs by calendar year among patients with at least one documented transportation need.

Calendar year	Total notes, N	Counts of transportation needs	Rates of transportation needs (%)
2016	2542	76	3.0
2017	2986	78	2.6
2018	3180	91	2.9
2019	3824	115	3.0
2020	4616	182	4.0
2021	5364	230	4.3
2022	5276	260	4.9

**Table 5.** Time between consecutive transportation needs.

Number of transportation need events	Patients (n=726), n (%)	Time between consecutive needs (days), median (IQR)	Time between the first encounter date (days), median (IQR)
1	566 (78.0)	—^a^	127 (21-595)
2	97 (13.4)	4 (0-67)	282 (15-776)
3	30 (4.1)	69 (5-161)	406 (142-1072)
4	13 (1.8)	42 (4-121)	512 (238-1148)
5	5 (0.7)	96 (69-156)	596 (312-1175)
6	7 (1.0)	182 (91-285)	810 (558-1215)
7	3 (0.4)	85.5 (69-145)	1353 (666.2-1845)
8	2 (0.3)	156 (154-181)	1663 (1354-2288)
9	3 (0.4)	5 (3-71)	1799 (1579-2044)

a Not available.

## Discussion

### Principal Findings

In this study, we developed a rule-based NLP algorithm to identify transportation needs from the free text of outpatient ophthalmology clinic encounters. Our model was trained and validated using human-labeled gold-standard datasets, and the NLP algorithm demonstrated a satisfactory performance with an *F*_1_-score of 0.860. Overall, we found that 0.6% (726/118,518) of patients had a transportation need that was identified from their ophthalmology clinic documentation.

The performance of our algorithm is comparable to the *F*_1_-score of 0.60 reported by Guevara et al [12], who used large language models to identify mention of transportation issues in oncology clinical notes. Their study focused on a similar patient population but used different methodologies, using fine-tuned Flan-T5 models and ChatGPT family models. Interestingly, despite the differences in approach, this study and the study by Guevara et al [12] demonstrated the feasibility of using NLP to identify mention of transportation needs from clinical documentation. Guevara et al [12] also noted that text-extracted SDoH identified significantly more patients with needs than structured data that use diagnostic codes, which aligns with our findings about the value of mining unstructured clinical notes.

Our model demonstrated higher precision than recall, which indicated a tendency to produce more false negatives than false positives, suggesting that this result may represent the lower limit of identified transportation needs. Our post-hoc evaluation revealed important limitations in recall when applied to notes with more diverse expressions of transportation difficulties. Transportation needs may be expressed through complex linguistic constructions, including negations (“cannot drive”), hypothetical language (“may have difficulty with transportation”), synonyms not included in our keyword list (eg, “mobility issues”), and implied inferences that require contextual understanding (eg, “patient’s vehicle was under repair”). These linguistic challenges are consistent with those identified in other NLP applications for SDoH text extraction from clinical notes [19]. The error analysis of false negatives in our post-hoc evaluation highlighted specific areas for improvement: handling negation patterns, detecting hypothetical language, expanding synonym recognition, and developing more sophisticated contextual understanding. These findings align with previous research on the challenges of extracting social needs information from clinical text [12,16].

### Comparison to Prior Work

Previous studies have explored using NLP to identify transportation needs within EHRs [12,16]. One group developed algorithms to detect housing, food, and transportation insecurity among patients in a large health care network. Their transportation algorithm demonstrated relatively low performance compared to the algorithms for housing and food insecurity, and its performance was also lower than that of our algorithm [16]. Another team created an algorithm to identify social needs among oncology patients, with lower performance for the transportation algorithm than other SDoH domains [12]. However, the transportation cases in their study are low, with only 41 in the training set and 6 in the testing set, which may limit the reliability of their algorithm.

Our unique focus on ophthalmology clinics presented distinct challenges in EHR data mining. Free text from ophthalmology clinic encounters often includes abbreviations and terminology that are highly specific to the field [21,22]. We found that refining our algorithm for identifying mention of social needs in ophthalmology was an important step in the process. It required collaboration between ophthalmologists and data scientists to ensure the algorithm did not mistakenly identify common ophthalmology terms, such as “difficulty driving” for cataract patients and the “transit” phase of fluorescein angiography—an ophthalmic dye-based imaging test—as transportation needs. The most common transportation terms identified from unstructured clinical notes were “due to transportation,” “transportation issues,” and “transportation difficulties.”

The 0.6% prevalence of transportation needs identified by our NLP algorithm is similar to the reported transportation needs hit rate of 1.3% reported from a social needs algorithm developed and tested on unstructured medical notes in another large health system [16]. Interestingly, the use of NLP algorithms to detect mention of social needs in health care settings may result in a lower hit rate than the use of structured EHR-based social needs questionnaires. For example, an EHR-based social needs screening questionnaire for 1696 primary care patients in Boston identified that 7% of screened patients reported a transportation need, which is much higher than the approximately 1% rate in the NLP-based studies [23]. Another reported the presence of transportation insecurity in 2% (3062/147,150) of patients [24]. Different clinical settings will have a variable proportion of underserved patients and unique HRSN, and differences may be apparent between primary care settings and subspecialty offices. Nonetheless, there does seem to be a pattern of a higher proportion of detected needs in survey studies than in NLP algorithms. For example, in our own patient population—encompassing some of the same cohort that underwent NLP assessment—5.1% (68/1340) of patients who completed an EHR-based social needs questionnaire reported a transportation need [25]. While the proportion of patients identified in structured questionnaires is higher than that in NLP algorithms, surveys depend on participant uptake, which may be low and subject to nonresponse bias [24]. Furthermore, screening surveys may increase the documentation demands on health care providers [16]. The vast amount of data available in unstructured clinical notes makes it a promising resource for automated EHR mining. While the percentage of identified social needs may be lower for NLP algorithms than survey-based assessments, the absolute number of patients identified can be much greater [26]. As such, a robust NLP model capable of detecting mentions of social needs during clinical encounters, along with their frequency, offers a valuable alternative for understanding and tracking these needs over time cost-effectively.

Patients with a transportation need identified by the algorithm were more likely to be older and of a non-Asian race, with no difference between sexes or between Black and White patients. Our study’s finding that older age is a risk factor for transportation insecurity is consistent with previous research [27]. While some studies have identified the female sex as a risk factor for transportation barriers to health care access [27], our findings align with other research indicating no significant difference between sexes [28]. Previous studies have demonstrated that racial and ethnic minority groups have higher rates of transportation insecurity [28]. Specifically, one study that used an NLP algorithm to extract SDoH text from the clinical notes of patients with cancer and opioid users found that transportation documentation was higher among Black patients [29]. Our study found no difference in transportation insecurity between Black and White patients. Possible explanations for this discrepancy could be a decreasing gap in travel burdens between Black and White Americans in recent years or a hesitance among minority patients to communicate with health care providers, which may result in the absence of this information in the unstructured clinical note in the EHR, but further study is warranted [30,31].

Knowledge about how a person’s social needs, particularly transportation needs, change over time remains limited. One study surveyed 228 participants about transportation difficulties at baseline and then reassessed them at either 3 or 6 months [32]. It found that 9% of the population initially reported transportation difficulties, and these needs remained largely stable over time. Only 6% to 8% reported improvement, and 5% reported worsening. The authors suggested that a 3- to 6-month follow-up may be sufficient for identifying changes in social risk [32]. In contrast, our study found that just 0.1% (n=117) of individuals were identified as having transportation needs at their first encounter, which was substantially lower than the findings of the previous study. This discrepancy may reflect differences in population characteristics; for example, our cohort had less racial diversity, and race is a known factor associated with travel-related disparities [33].

Among individuals identified as having transportation needs, 78.0% (566/726) had only one documented need, with a median of 127 (IQR 21-595) days from their first encounter. For those with 2 documented needs, the median interval was 282 (IQR 15-776) days since the initial visit. While these results do not clarify whether the transportation needs identified by the NLP algorithm precisely reflect the timing of the actual need, it is possible that the observed delay stems from a growing sense of trust between patients and their physicians, which may lead patients to disclose their needs only after several visits [34]. Regardless of the cause, these findings suggest that a 3- to 6-month follow-up period may be insufficient to capture meaningful changes in transportation needs among patients facing such barriers.

### Potential Interventions

Identification of transportation needs by NLP algorithms may facilitate the implementation of actionable interventions to address transportation barriers. Medical transportation resources are readily available through health insurance and county-based programs, but many patients are not aware that they are eligible for these benefits. For example, at our own institutions, 50 out of 52 ophthalmology patients with transportation needs who were referred to our patient navigator program for resources had their transportation issues resolved by enrolling in a transportation assistance program [8]. The biggest challenge in assisting patients with transportation resources is identifying the patients with transportation needs. By implementing a transportation-oriented NLP algorithm in clinical practice, institutions can identify patients who are at high risk of transportation insecurity and contact them in advance of an upcoming appointment to assess for transportation challenges and to offer resources. In fact, patients with missed appointments have expressed support for outreach with resources to maintain eye care [5], and the implementation of an NLP algorithm to flag patients with needs could greatly facilitate resource allocation to help vulnerable patients maintain access to care.

### Limitations

To our knowledge, this study is the first to apply an NLP-based transportation analysis to a corpus of ophthalmology clinic notes from the EHR of a large health care system. Our algorithm identified 726 ophthalmology patients with a transportation need, demonstrating its potential to detect transportation insecurity without adding to health care providers’ workloads. Our study does have several limitations. First, our algorithm was validated and applied to a population local to western Pennsylvania, which may not be nationally representative [35]. In particular, the preponderance of White patients in our dataset may limit our ability to detect racial disparities in transportation needs for patients of the Black race or Hispanic ethnicity. Future work should examine the performance of our algorithm using ophthalmology clinic notes from other geographic settings, particularly those that represent greater racial and ethnic diversity.

Second, the true prevalence of transportation insecurity among our patient population is unknown, making it difficult to compare our findings to actual rates. To assess our findings in the context of an unknown true prevalence of transportation insecurity, we conducted a post-hoc analysis with broad transportation terms to examine for false negatives. While the precision (1.00) of the algorithm in this post-hoc analysis remained high, the recall (0.71) was less robust, indicating that some true transportation needs are missed by the algorithm in an attempt to mitigate false positives. The implications of our post-hoc analysis suggest that our results are probably underreporting the true prevalence of transportation needs in our population. Specifically, our model’s higher precision than recall suggests that we likely present a conservative estimate of identified transportation needs in our patient population.

A third limitation of this study is the examination of transportation needs without the consideration of other concomitant, and likely interrelated, HRSN. For this study, we used “transportation needs” as an umbrella term to mine the EHRs, but transportation insecurity has many components and can overlap with other SDoH domains, such as financial insecurity [36]. Patients who express financial difficulties, for instance, may also have difficulty with transportation, but these patients would not be identified by our algorithm unless their needs are documented with transportation-specific language. Therefore, our detected prevalence of transportation needs may again be lower than the true burden faced by the patient cohort. Future work should develop algorithms to identify other SDoH domains in the context of eye care encounters. It is important to explore the different facets of transportation and how they interact with other social needs to identify specific transportation challenges and direct patients to the appropriate services to address these issues.

As a fourth limitation, relevant factors such as socioeconomic status, education level, and residence information were not included in the data source. Absence of these data limits our ability to gain further insights into baseline characteristics that may warrant attention. Future work should incorporate these sociodemographic components, which could facilitate predictive modeling of a patient’s risk of transportation needs.

### Future Directions

External validation of this model using data from other health care systems across the United States is necessary to test its generalizability. For implementation into clinical practice, further research is needed to assess how health care teams may engage with potential notifications in the EHR about transportation needs identified by the NLP algorithm.

### Conclusions

Our NLP algorithm successfully identified previously unknown transportation needs from unstructured ophthalmology clinic notes in the EHRs. The algorithm demonstrated reliable identification of transportation insecurity; thus, it offers an additional method for leveraging large amounts of available data to screen for transportation needs among ophthalmology patients, without adding to the documentation burden on health care providers. Our findings indicate age disparities in transportation needs within our patient population, with older patients indicating a greater burden of transportation needs. In addition to survey-based social needs screening, NLP can identify mention of social needs from EHR text, thus potentially facilitating referrals to transportation resources for vulnerable ophthalmology patients.

## Supplementary material

10.2196/69216 Multimedia Appendix 1 Post-hoc analyses.

## Abbreviations

AI: artificial intelligence
EHR: electronic health record
HRSN: health-related social needs
NLP: natural language processing
OR: odds ratio
SDoH: social determinants of health
