# Supplementary material for: Identifying Transportation Needs in Ophthalmology Clinic Notes Using Natural Language Processing: Retrospective, Cross-Sectional Study
Source: JMIR Med Inform. 2025 Sep 5;13:e69216. doi: 10.2196/69216 (PMC12413321; doi:10.2196/69216)
Supplement: Multimedia Appendix 1 [file medinform-v13-e69216-s001.docx]

Supplemental Material:

We performed a post-hoc evaluation on 200 clinical notes pre-filtered for the token “transport*” to broadly capture any mention of transport-related barriers. Within this set, our binary classifier achieved 85.5% accuracy overall. As shown in the confusion matrix, all 101 “No Issue” notes were correctly identified (precision & recall = 1.00), while of the 99 true “Transportation Issue” notes the model only caught 70 (precision = 1.00, recall = 0.71, F₁ = 0.83). Mapping the 29 false negatives back to Fu et al. (2024)’s taxonomy reveals that roughly half are Contextual Errors (Dim 2)—chiefly Negation (2.2.4) and Possible/Probable Language (2.2.5)—and half are Linguistic Errors (Dim 3), dominated by Synonym mismatches (3.3.4) and Implied Inference (3.3.2). To boost recall without sacrificing our perfect precision, we therefore recommend augmenting the model with stronger negation/hypothetical-language handling and expanded synonym/implication lexicons.


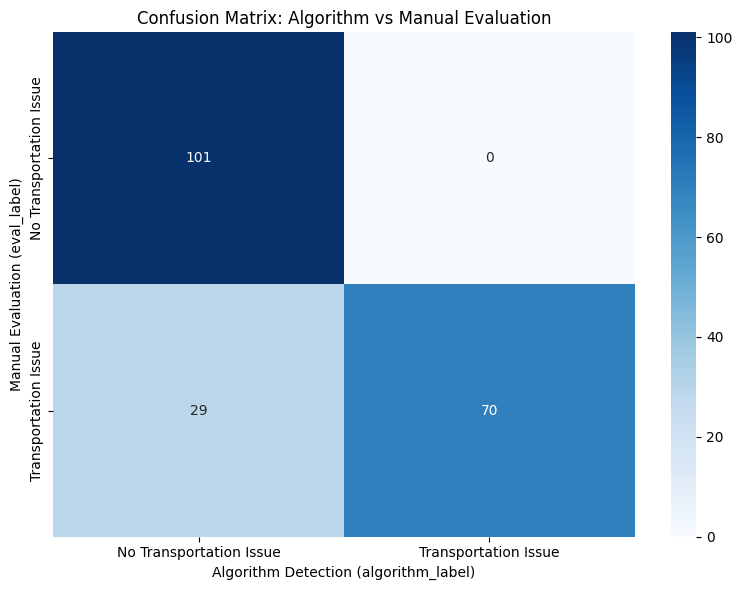


Classification Report:

precision recall f1-score support

No Issue 0.78 1.00 0.87 101

Transportation Issue 1.00 0.71 0.83 99

accuracy 0.85 200

macro avg 0.89 0.85 0.85 200

weighted avg 0.89 0.85 0.85 200

Metrics Summary:

Accuracy: 0.855

Precision: 1.000

Recall: 0.707

F1 Score: 0.828

Number of disagreements: 29

Total samples: 200

Agreement rate: 0.855

Broader keywords:

A stratified sampling methodology was implemented using six contextual categories of keywords including appointment context (missed appointments, scheduling issues), mobility context (physical limitations), assistance context (family/caregiver help), vehicle context (transportation modes), circumstantial context (weather, financial barriers), and location context (distance factors). The approach created a composite scoring system ranging from 0-6 points based on the number of pattern categories matched per document, with stratified sampling targeting 200 documents across high-priority (60 documents with 3+ categories), medium-priority (60 documents with 2 categories), low-priority (50 documents with 1 category), and random baseline (30 documents with 0 categories) groups.

import pandas as pd

import numpy as np

import re

# Set random seed for reproducibility

np.random.seed(2025)

# Define selection patterns

selection_patterns = {

'appointment_context': [

'late', 'delay', 'miss', 'cancel', 'reschedule',

'postpone', 'early', 'time', 'arrived', 'arrival',

'wait', 'show up', 'came', 'appointment', 'visit',

'schedule', 'appt', 'no show', 'didn\'t come'

],

'mobility_context': [

'wheelchair', 'walker', 'mobility', 'ambulate',

'walk', 'cane', 'scooter', 'gait', 'steps',

'stairs', 'elevator', 'accessible', 'disability',

'handicap', 'fall risk', 'unsteady', 'assistance device',

'transfer', 'ambulatory'

],

'assistance_context': [

'help', 'assist', 'neighbor', 'family', 'friend',

'son', 'daughter', 'spouse', 'wife', 'husband',

'relative', 'companion', 'caregiver', 'someone',

'anybody', 'support', 'accompany', 'brought',

'dropped off', 'picked up', 'took', 'gave ride'

],

'vehicle_context': [

'car', 'vehicle', 'drive', 'repair', 'broken',

'bus', 'taxi', 'uber', 'lyft', 'ride', 'transport',

'transportation', 'van', 'truck', 'parking',

'traffic', 'gas', 'fuel', 'distance', 'miles',

'insurance', 'accident', 'breakdown', 'tow'

],

'circumstantial_context': [

'weather', 'rain', 'snow', 'ice', 'storm',

'due to', 'because', 'since', 'caused by',

'difficulty', 'problem', 'issue', 'challenge',

'unable', 'couldn\'t', 'can\'t', 'cannot',

'financial', 'money', 'cost', 'afford', 'expensive'

],

'location_context': [

'far', 'distance', 'miles', 'minutes', 'hours',

'rural', 'remote', 'city', 'downtown', 'suburb',

'home', 'facility', 'clinic', 'hospital', 'office',

'get to', 'reach', 'access', 'available'

]

}

def contains_pattern(text, patterns):

if pd.isna(text):

return False

text_lower = str(text).lower()

return any(pattern in text_lower for pattern in patterns)

text_column = 'NOTE_TEXT'

for category, patterns in selection_patterns.items():

sample_source[f'has_{category}'] = sample_source[text_column].apply(

lambda x: contains_pattern(x, patterns)

)

sample_source['pattern_score'] = (

sample_source['has_appointment_context'].astype(int) +

sample_source['has_mobility_context'].astype(int) +

sample_source['has_assistance_context'].astype(int) +

sample_source['has_vehicle_context'].astype(int) +

sample_source['has_circumstantial_context'].astype(int) +

sample_source['has_location_context'].astype(int)

)

# Stratified sampling

samples = []

# 1. High-priority samples (documents matching multiple patterns) - 60 documents

high_priority = sample_source[sample_source['pattern_score'] >= 3]

if len(high_priority) >= 60:

samples.append(high_priority.sample(n=60, random_state=2025))

else:

samples.append(high_priority)

remaining_high = 60 - len(high_priority)

# 2. Medium-priority samples (documents matching 2 patterns) - 60 documents

medium_priority = sample_source[sample_source['pattern_score'] == 2]

if len(medium_priority) >= 60:

samples.append(medium_priority.sample(n=60, random_state=2025))

else:

samples.append(medium_priority)

remaining_medium = 60 - len(medium_priority)

# 3. Low-priority samples (documents matching 1 pattern) - 50 documents

low_priority = sample_source[sample_source['pattern_score'] == 1]

if len(low_priority) >= 50:

samples.append(low_priority.sample(n=50, random_state=2025))

else:

samples.append(low_priority)

remaining_low = 50 - len(low_priority)

# 4. Random samples from remaining documents - 30 documents

no_pattern = sample_source[sample_source['pattern_score'] == 0]

if len(no_pattern) >= 30:

samples.append(no_pattern.sample(n=30, random_state=2025))

else:

samples.append(no_pattern)

# Combine all samples

final_sample = pd.concat(samples, ignore_index=True)

columns_to_drop = [col for col in final_sample.columns if col.startswith('has_')]

final_sample_clean = final_sample.drop(columns=columns_to_drop, errors='ignore')

print(f"Final sample size: {len(final_sample_clean)}")

print("\nSampling distribution:")

print(final_sample['pattern_score'].value_counts().sort_index())

# Save the sample

final_sample_clean.to_csv('0516-transportation_sample_200.csv', index=False)

Reference:

Sunyang Fu, Liwei Wang, Huan He, Andrew Wen, Nansu Zong, Anamika Kumari, Feifan Liu, Sicheng Zhou, Rui Zhang, Chenyu Li, Yanshan Wang, Jennifer St Sauver, Hongfang Liu, Sunghwan Sohn, A taxonomy for advancing systematic error analysis in multi-site electronic health record-based clinical concept extraction, *Journal of the American Medical Informatics Association,* Volume 31, Issue 7, July 2024, Pages 1493–1502, https://doi.org/10.1093/jamia/ocae101
